# Clinical Evaluation of an Affordable Qualitative Viral Failure Assay for HIV Using Dried Blood Spots in Uganda

**DOI:** 10.1371/journal.pone.0145110

**Published:** 2016-01-29

**Authors:** Sheila N. Balinda, Pascale Ondoa, Ekwaro A. Obuku, Aletta Kliphuis, Isaac Egau, Michelle Bronze, Lordwin Kasambula, Rob Schuurman, Nicole Spieker, Tobias F. Rinke de Wit, Cissy Kityo, ART–A consortium

**Affiliations:** 1 Joint Clinical Research Center, P.O. Box 10005, Kampala, Uganda; 2 Amsterdam Institute for Global Health and Development, Department of Global Health, Academic medical Center, Trinity C Building, Pietersbergweg 17, 1105 BM, Amsterdam, the Netherlands; 3 PharmAccess International, Amsterdam, Trinity Building C Pietersbergweg 17, 1105 BM, Amsterdam, the Netherlands; 4 Wits Health Consortium, University of the Witwatersrand, 1 Jan Smuts Avenue, Braamfontein 2000, Johannesburg, South Africa; 5 University Medical Centre Utrecht, P.O. Box 80125, 3508 TC, Utrecht, the Netherlands; George Washington University, UNITED STATES

## Abstract

**Background:**

WHO recommends regular viral load (VL) monitoring of patients on antiretroviral therapy (ART) for timely detection of virological failure, prevention of acquired HIV drug resistance (HIVDR) and avoiding unnecessary switching to second-line ART. However, the cost and complexity of routine VL testing remains prohibitive in most resource limited settings (RLS). We evaluated a simple, low–cost, qualitative viral–failure assay (VFA) on dried blood spots (DBS) in three clinical settings in Uganda.

**Methods:**

We conducted a cross–sectional diagnostic accuracy study in three HIV/AIDS treatment centres at the Joint Clinical Research Centre in Uganda. The VFA employs semi-quantitative detection of HIV–1 RNA amplified from the LTR gene. We used paired dry blood spot (DBS) and plasma with the COBASAmpliPrep/COBASTaqMan, Roche version 2 (VL_ref_) as the reference assay. We used the VFA at two thresholds of viral load, (>5,000 or >1,000 copies/ml).

**Results:**

496 paired VFA and VL_ref_ results were available for comparative analysis. Overall, VFA demonstrated 78.4% sensitivity, (95% CI: 69.7%–87.1%), 93% specificity (95% CI: 89.7%–96.4%), 89.3% accuracy (95% CI: 85%–92%) and an agreement kappa = 0.72 as compared to the VL_ref_. The predictive values of positivity and negativity among patients on ART for >12 months were 72.7% and 99.3%, respectively.

**Conclusions:**

VFA allowed 89% of correct classification of VF. Only 11% of the patients were misclassified with the potential of unnecessary or late switch to second–line ART. Our findings present an opportunity to roll out simple and affordable VL monitoring for HIV–1 treatment in RLS.

## Introduction

HIV/AIDS remains one of the world’s critical public health challenges with 36.9 million people living with HIV and 1.2 million AIDS–related deaths at the end of 2014 [1,2]. Sub-Saharan Africa, which represents 2.1% of the global Gross domestic product (GDP) [3], is disproportionately affected and holds 70% (25.8 million) of the world’s HIV/AIDS burden [1,2]. Nonetheless, recent evidence demonstrates unprecedented milestones in the global AIDS response with a decline in the number of new infections and deaths [1,2].

Indeed there has been an exponential increase in ART coverage since 2003, with 41% (15 million people) of eligible Persons Living With HIV/AIDS (PLWHAs) accessing therapy in sub-Saharan Africa as of march 2015 [1,2]. This rapid expansion in ART coverage creates an urgent need for a strengthened laboratory support network for early diagnosis of HIV, timely monitoring of HIV treatment and early detection of resistance due to failing ART regimens.

Despite existing evidence and the 2013 WHO recommendations that VL testing is crucial in predicting clinical outcomes among PLWHAs taking ART [4], implementation considerations are inhibiting the scale up of this technology in sub–Saharan Africa. A recent report indicates that less than 20% of African patients on ART have access to a VL testing [5]. Costs and complexity are often prohibitive due to expensive VL detection equipment, the need for human resource training and laboratory infrastructure as well as operational challenges in sample collection, transport, storage and processing. Notwithstanding, the WHO makes a strong recommendation that VL is the preferred monitoring approach to diagnose and confirm ART failure.

A previous systematic review published in 2009 indicated that DBS are a practical alternative specimen source to liquid plasma for HIV testing, in terms of a stable specimen matrix, ease of sample collection, storage and transportation [6]. We have previously reported the development of a qualitative VFA which is simple optimizes an open platform and is compatible with finger or heel prick DBS collection [7]. This assay was specifically designed to function as a tie-breaker for a subsequent HIV–1 drug resistance test [8]. In the current paper, we report the performance of this VFA as a screening tool to determine treatment failure using DBS among PLWHAs.

## Methods

### Ethical considerations

We obtained ethical clearance for the use of patient sample material was obtained through the Ethics review committees of JCRC, the Uganda National Council of Science &Technology (UNCST), and the Academic Medical Center of the University of Amsterdam, Netherlands. All adult participants, and parent(s) or guardian(s) of all eligible children provided written informed consent. Children above the age of 8 years who were aware of their HIV status provided written informed assent.

### Setting

The Joint Clinical Research Centre (JCRC) is a pioneer HIV/AIDS care, research and training institute in Africa founded in 1991, (www.jcrc.org.ug). The JCRC operates a network of 7 Regional Centres of Excellence in Uganda, which provide comprehensive AIDS care and advanced laboratory tests including VL measurements. Mbale, Fort Portal and Kampala have high patient loads, are geographically representative and therefore selected for this study. In 2012, the 3 centres attended to at least 10,000 PLWHAs, whilst over 100,000 PLHWAs have ever accessed services at JCRC sites countrywide.

### Design

In a cross–sectional diagnostic accuracy study between 2012 and 2013, we compared the performance of the index VFA to the standard reference VL test (VL_ref_). We report these findings in line with the Standards for Reporting Diagnostic Accuracy Studies statement (STARD) [9].

### Participants, sampling and sample size estimation

We collected DBS samples from HIV-1 positive individuals participating in the PASER-M program [10] (36 month follow-up) and MARCH[11] studies (baseline, 6, 12 months). Using Burderer’s formula [12] we estimated that a sample size of at least 490 would be sufficient to test for diagnostic accuracy, anticipating VFA sensitivity of 85%, a true virological failure of 10% and 10% precision for a 95% Confidence Interval. These parameters were informed by our previous studies, [7,13].

### Sample storage and Transportation

We stored DBS and plasma samples at -20°C and -80°C respectively, tested on site using robust affordable real-time PCR instruments (Mini-opticon). We transported plasma samples in dry-ice to the reference laboratory in Kampala for the reference testing (VL_ref_) using the COBASAmpliPrep/ COBASTaqManSystem,Roche version 2.

### Nucleic Acid Isolation

We described details of the laboratory procedures in our previous work published elsewhere [7]. We performed nucleic acid isolation from DBS using the QIAamp RNA kit (Qiagen GmbH, Germany), according to manufactures instructions. Briefly, we excised DBS samples by hand using scissors and placed two of the spots in lysis buffer and incubated at room temperature with gentle rotation for 30 min, subsequent washes and a final elution of 50μl.

### Reverse Transcription

We conducted the reverse transcription as described elsewhere [6]. In short, we used the eluent comprising both HIV-1 RNA and internal control RNA, and reverse transcribed using the TaqMan Real-time PCR system Random Hexamers RT kit (Life Technologies, Foster City, CA) according to the manufacturer’s instructions.

### Real-time PCR

We amplified HIV-1 and IC cDNA fragments in multiplex format using 300nM primer *EMC-forward* (5’-TGACCACGCCACCGC-3’), 900nM primer *EMC-reverse* (5’-TAAAGATTTCCCTTGCCCCG-3’), 100nM probe *EMC-VIC* (5’-TGTGAGCCAGTCGTGATTGTGCTCC-3’), 300nM forward primer *LTR S4* (5’-*AAGCCTCAATAAAGCTTGCCTTGA*-3’; HXB2 nt520-543), a mixture of 600nM HIV-LTR reverse primers *3’UNI-KS-6* (5’- *GAGGGATCTCTAGTTACCAGAGTCACA*-3’; HXB2 nt574-600) and *3’UNI-KS-6-AG* (5’- *GAGGGATCTCTAGTTACCAGAGTCCTA*-3’; ssssHXB2 nt574-600) and 100nM MGB probe *HIV-LTR-FAM* (5’- TAGTGTGTGCCCGTCTG -3’; HXB2 nt554-570); using a MiniOpticon Real-Time PCR Detection System (BioRad, Hercules, CA). This system included a temperature profile allowing for dUTP/UNG decontamination; 50°C for 2 minutes; 95°C for 10 minutes; 45 cycles of 95°C for 15 seconds and 60°C for 1 minute. We validated the result of a clinical sample when the positive and negative controls, as well as the internal control, met the acceptance criteria.

### HIV-1 Viral subtype

We determined the HIV-1 viral subtype in our laboratories in Amsterdam (based on baseline HIVDR sequence data) using REGA HIV-1 subtyping tool version 3.0.

### Outcome measures

These included the following measures of diagnostic accuracy: sensitivity, specificity, negative predictive value (NPV), positive predictive value (PPV), area under curve (AUC), kappa agreement, likelihood ratios (LR) and Diagnostic Odds Ratios (DOR).

### Data management and statistical considerations

We captured clinical and laboratory data onsite (single data entry) during routine patient visits, entered these into a central database and controlled for quality using query systems. We used Stata® version 11.2 (College Station, Texas, USA) for analysis. We tabulated patient characteristics into frequencies, proportions and appropriate measures of central tendency. Thereafter, we employed the Stata® commands “roctab”, “roccomp”, “rocgold” and “diagti” to estimate the diagnostic accuracy outcome measures compared to the reference standard, with 95% confidence intervals where appropriate. We used two definitions of viral failure as greater than 5,000 viral copies/mL (in line with the initial WHO cut–off for switching to second-line antiretroviral therapy) and greater than 1000 copies (in line with the most recent WHO guidelines [4]). We conducted sensitivity analyses by varying age strata (children, adults), duration on antiretroviral therapy (<6 versus 12–36 months), HIV-1 viral subtype and study site (Kampala, Fort Portal and Mbale). Children tend to have higher VL and their adherence patterns largely depend on their adult guardians [14]; duration on ART reflects the durability of treatment effect, whilst HIV-1 viral subtype may vary the treatment effect and user dependence may explain variation of assay performance across the study sites. We computed costs for equipment and kits from multiple sources including local facility procurement records, published studies or conference presentations, or websites of the manufacturer (http://www.bio-rad.com/ and http://www.roche.com/). We obtained salary information for Laboratory Technicians from the Uganda Public Service Single Spine Structure. We converted all costs to 2015 US dollars, (One US dollar ~ Uganda shillings 2,940).

## Results

### Clinical and socio-demo graphic characteristics of study participants

Four hundred and ninety six samples of paediatric (MARCH) and adult (PASER) HIV patients on ART were consecutively submitted for VL testing. We tested 168 (33.9%) from the MARCH cohort with a mean age of 5.6 years (SD: 3.5), of whom 54% were girls. The PASER cohort constituted 328 adults (66.2%) with a mean age of 38.2 (9) and majority were female (57.6%). The 496 samples were geographically from Mbale (147), Fort Portal (151) and Kampala (198). One hundred twenty four baseline samples were included and 372 participants (75%) had been on ART for at least 6 months allowing for assessment of VFA. Subtypes A (54.5%) and D (33.2%) were the predominant HIV–1 viral subtypes. Details of baseline characteristics are in Table 1.

**Table 1:** Characteristics of the MARCH and PASER cohorts.

Characteristic	MARCH (n = 168)	PASER (n = 328)	Total (n = 496)
Age (years, mean, sd)	5.6 (3.5)	38.2 (9.3)	25.8 (16.8)
Sex (Female)	91 (54.2)	189 (57.6)	280 (56.4)
Orphan	115 (67.3)	n/a	115
CD4+ (%, median, IQR)	18 (12.6–25.4)	26 (19.8–35)	24 (16–32)
*§*CD4+ (absolute, median, IQR)	649 (318–948)	346 (228–521)	403 (255–628)
Viral Load (log10, median, IQR)	4.6 (3.6–5.2)	1.3 (1.3–2.3)	3.6 (1.3–4.8)
ART regimen (2^nd^ line)	51 (30.4)	missing	51
*Subtype			
A	82 (49.7)	158 (57.5)	240 (54.5)
D	39 (23.6)	107 (38.9)	146 (33.2)
C	5 (3.0)	7 (2.5)	12 (2.7)
Recombinant	19 (11.5)	0 (0.0)	19 (4.3)
Complex	10 (6.1)	0 (0.0)	10 (2.3)
Other	10 (6.1)	3 (1.1)	13 (3.0)

All figures in parentheses are % unless stated otherwise

§–CD4+ cell counts at time of sampling for Viral Load measurements

*–Data for HIV-1 subtype was available for 440 samples

### Overall performance of the Viral Failure Assay

**Fig 1:**
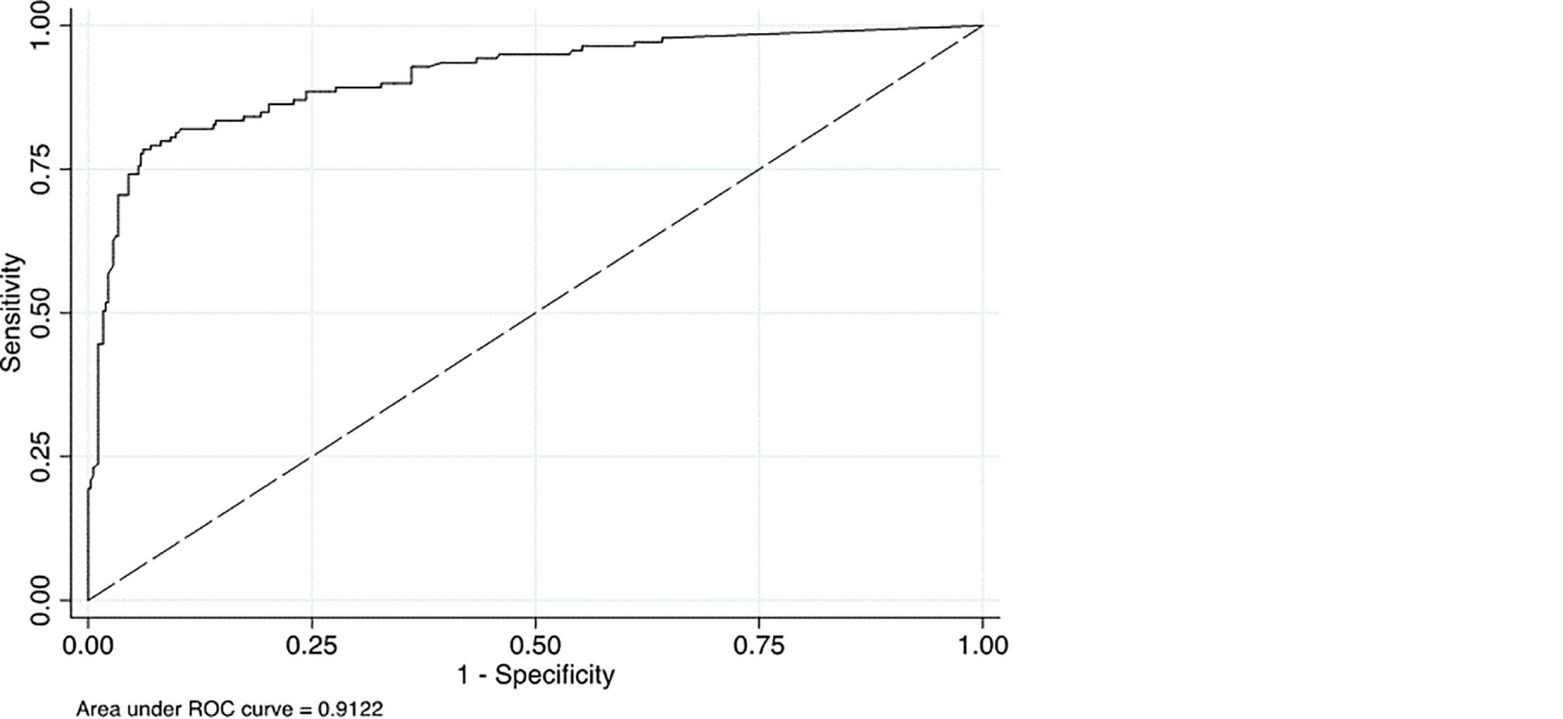
Receiver Operating Characteristics Curve for VFA compared to Cobas-Ampliprep. NB: AUC–Area under curve denotes the area in the graph below Receiver Operating Characteristic (ROC) curve, comparing the true positive to the true negative values. The true positive rate (sensitivity) is plotted in function of the false positive rate (100 –specificity) for different cut–off points. Each point on the ROC curve represents a sensitivity/specificity pair corresponding to a particular decision threshold. A test with perfect discrimination (no overlap in the two distributions) has a ROC curve that passes through the upper left corner (100% sensitivity, 100% specificity). Therefore the closer the ROC curve is to the upper left corner, the higher the overall accuracy of the test. An area of 1 represents a perfect test whilst 0.5 is a worthless test.

The prevalence of VL ≥ 5,000 copies/mL, our primary cut-off point, was 27.0% (95% CI: 22.9%–32.9%) as determined by the gold standard VLref assay and depicted in Table 2. VFA compared to the VL_ref_ demonstrated 78.4% sensitivity, (95% CI: 69.7%–87.1%), 93% specificity (95% CI: 89.7%–96.4%) and 89.3% accuracy (95% CI: 85%–92%). The AUC was 0.91 (95% CI: 0.88–0.94) whilst the kappa agreement between VFA and VL_ref_ was 72.2% (95% CI: 61.1%–83.4%). The predictive value of positivity and negativity were 81.3%(95% CI: 72.9%–89.8%%) and 91.8% (95% CI: 88.2–95.4%) respectively. The likelihood ratio for a positive test was 11.3 (95% CI: 6.87–18.5) while that for a negative test was 0.23 (95% CI: 0.16–0.35). The Diagnostic Odds Ratio was 48.5 (95% CI: 13.1–84.0). The Area Under Curve (AUC) was 0.91 (Fig 1).

**Table 2:** VFA characteristics by ART status, age, VF threshold and HIV-1 subtype for >5000 copies/mL threshold.

Category	N (%)	Sensitivity	Specificity	Accuracy	PPV	NPV
**ART status**						
Naive	124 (25)	0.794	0.545	0.750	0.890	0.364
>6 months	372 (75)	0.757	0.955	0.941	0.651	0.973
12–36 months	342 (66)	0.889	0.981	0.968	0.727	0.993
**Age groups**						
Children	168 (33.9)	0.769	0.604	0.726	0.830	0.509
Adults	328 (66.1)	0.889	0.981	0.979	0.762	0.993
**Subtype**						
A	240 (54.5)	0.814	0.923	0.869	0.814	0.923
D	146 (33.2)	0.75	0.953	0.902	0.844	0.919
**VF threshold**						
> 1000 cp/mL	164 (33.1)	0.957	0.386	0.574	0.434	0.949
> 5000 cp/mL	134 (27.0)	0.784	0.930	0.890	0.813	0.918

PPV: Positive predictive value; NPV: Negative Predictive value

### Sensitivity analyses

The prevalence of VL ≥ 1,000 copies/mL, our primary cut-off point, was 33.1%. At this lower HIV treatment viral failure threshold of 1,000 cp/mL, we recorded a higher yield of 95.7% sensitivity (95% CI: 91.8%–99.7%), but lower specificity of 38.6% (95% CI: 32–45%) and 57.4% accuracy (95% CI: 51.9%–63%) for the VFA as compared to VL_ref_. These results are shown in Table 3. The AUC was 67.2% (95% CI: 64.2%–70.3%), whilst the kappa agreement between VFA and VL_ref_ was 26.3% (95% CI: 18.2%–34.4%). The predictive value of positivity and negativity were 43.4% (95% CI: 36.9%–49.9%) and 94.9% (95% CI: 90.1%–99.6%) respectively. The likelihood ratio for a positive test was 1.56 (95% CI: 1.39–1.76) while that for a negative test was 0.11 (95% CI: 0.04–0.28). The Diagnostic Odds Ratio was 14.1 (95% CI: -8.2–28.3).

**Table 3:** VFA characteristics by ART status, age and HIV-1 subtype for >1000 copies/mL threshold.

Category	N (%)	Sensitivity	Specificity	Accuracy	PPV	NPV
**ART status**						
Naive	124 (25)	0.973	0.273	0.911	0.932	0.500
>6 months	372 (75)	0.922	0.390	0.463	0.193	0.969
12–36 months	342 (66)	0.920	0.405	0.445	0.113	0.984
**Age groups**						
Children	168 (33.9)	0.964	0.200	0.828	0.848	0.545
Adults	328 (66.1)	0.920	0.405	0.445	0.113	0.984
**Subtype**						
A	240 (54.5)	0.975	0.369	0.576	0.444	0.967
D	146 (33.2)	0.951	0.431	0.580	0.402	0.957

PPV: Positive predictive value; NPV: Negative Predictive value

**Fig 2:**
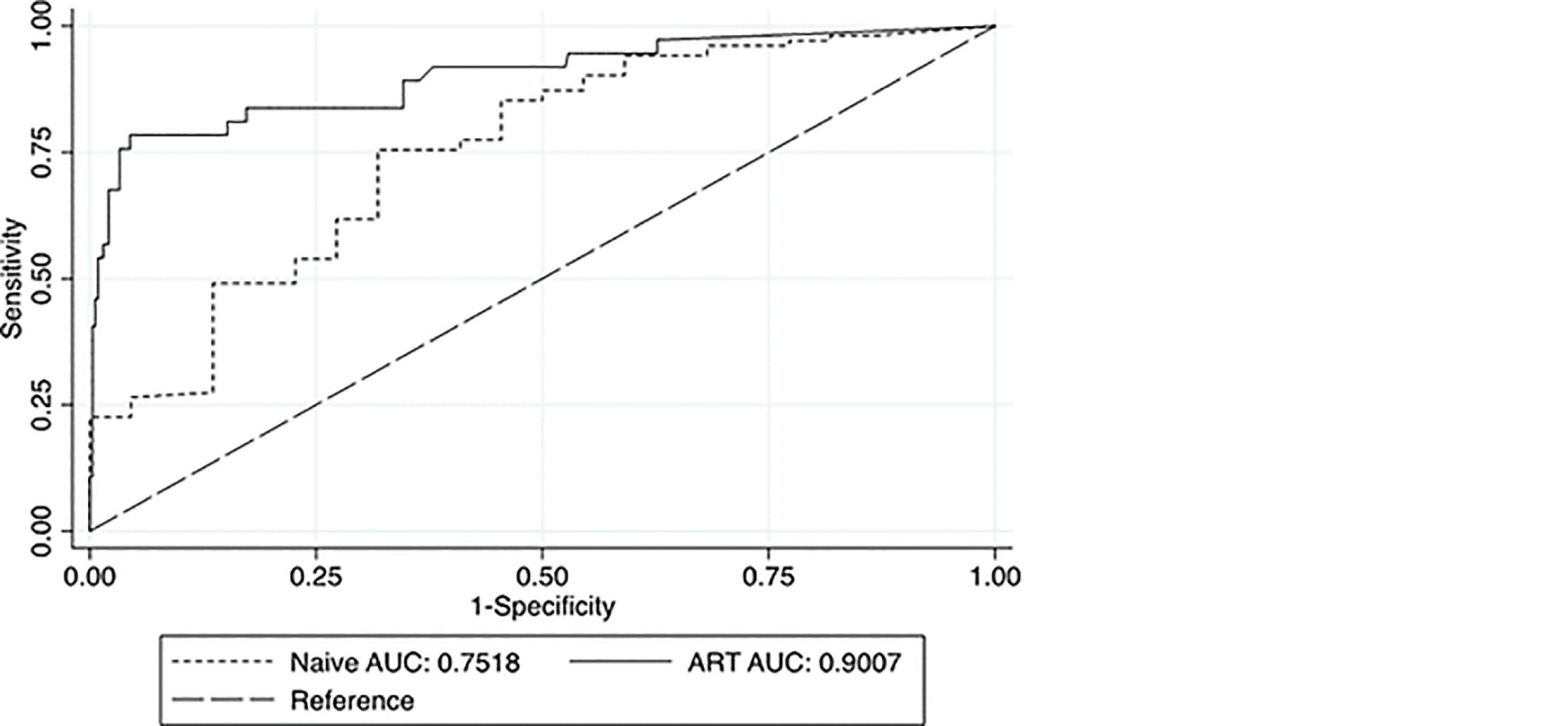
Receiver Operating Characteristics Curve for VFA comparing ART–naïve and treated PLWHAs (VL threshold ≥ 5,000 cp/mL).

Three hundred and seventy two (75%) and 342 (68.9%) participants had been on ART for at least 6 months and 12–36 months respectively. At the threshold of VL ≥ 1,000 copies/mL, the predictive values of positivity and negativity among patients on ART for > 6 and > 12–36 months were PPV, 65.1% (95% CI: 49.1–79) versus 72.7% (95% CI: 49.8–89.3) and NPV, 97.3% (95% CI: 94.9–98.7) versus 99.3% (95% CI: 97.7–99.9) respectively. The VFA Diagnostic Odds Ratios in these clinical ART groups were 77.1 (95% CI: 23.4–253.5) and 253 (95% CI: 48.2–1327.3) correspondingly. One hundred and twenty four samples were naïve to ART and the AUC for this sub–population was 0.75 compared to 0.90 for those exposed to ART (Fig 2).

The VFA performance in children compared to adults demonstrated less favourable performance characteristics: 76.9% (95% CI: 68.3–84.) versus 88.9% (95% CI: 65.3–98.6) sensitivity; 60.4% (95% CI: 45.3–74.2) versus 98.1% (95% CI: 95.8–99.3) specificity, and 72.6% (95% CI: 64.0–81.2) versus 97.9% (95% CI: 95.9–99.9) accuracy, (Table 2).

The VFA had a better sensitivity for HIV-1 viral subtype A at 81.4% compared to 75% for subtype D, at a cut-off of >5,000 copies/ml. When we lowered the cut off to >1,000 copies/ml, the sensitivity results were similar at 97.5% (subtype A) and 95.1% (subtype D).

Finally, VFA test accuracy ranged from 87.9% in Kampala, 88.4% in Mbale, to 92.1% for the Fort Portal site (Table 4).

**Table 4:** Performance of VFA across the three study sites.

Study site	Samples tested	True Negative	True Positive	Correctly classified	False Negative	False Positive	Incorrectlyclassified
Kampala	198	132 (0.67)	42 (0.21)	174 (0.88)	12 (0.06)	12 (0.06)	24 (0.12)
Fort Portal	151	119 (0.79)	20 (0.13)	39 (0.92)	5 (0.03)	7 (0.05)	12 (0.08)
Mbale	147	83 (0.57)	47 (0.32)	130 (0.88)	13 (0.09)	4 (0.03)	17 (0.12)
Combined	496	334 (0.67)	109 (0.22)	443 (0.89)	30 (0.06)	23 (0.05)	53 (0.11)

NB: The two tests are compared at the WHO cut-off: <5,000 cp/mL (not eligible for HIV-DR testing) and >5000 cp/mL eligible for HIV-DR testing)

### Costing analysis

In Table 5, we report that the estimated capital costs for equipment of VFA was US$ 22,000 compared to US$ 200,000 for the reference standard. There were 250 and 48 tests per kit for the VFA method and the reference test estimated at US$ 6,968 and US$ 2,098 respectively. This translates to a unit cost of US$ 27.9 and US$ 49.7. Although the VFA method allows for up to 48 tests per run, logistical considerations permit about 24 per Laboratory Technician. Thus, the cost per run of VFA is US$ 683.2 (US$ 1,366.4 for 48 tests) compared to US$ 2,105.8 for the reference standard.

**Table 5:** Cost estimates for ART-A compared to the reference HIV-1 viral load test.

^#^Cost variable	ART-A (US$)	Reference test (US$)
Test kit	6,968.1	2,098
Number of tests per kit	250	48
Unit cost of each test	27.9	43.7
Turn-around-time (hours)	7.5	4.5
Number of tests per run	*24 (48)	48
Salary (Laboratory Technician)	(898,000), 305.4	(898,000), 305.4
Labor per run (TAT * salary)	13.63	8.18
Cost per run	683.2 (1,366.4)	2,105.8
^§^Equipment	22,000	200,000

^§^Equipment for ART–A is the MiniOpticon real-time PCR detection system #359–1592, and a laptop computer whilst the reference standard is COBAS AmpliPrep and COBAS TaqMan® Systems

^#^ All costs were converted to 2015 US$. Costs for equipment and kits were computed from multiple sources including local facility procurement records, published studies or conference presentations, or from the website of the manufacturer (http://www.bio-rad.com/ and http://www.roche.com/); salary for Laboratory Technician (UGX 898,000) was obtained from the Uganda Public Service Single Spine Structure; the cost per run = (labour per run)*(unit cost of each test)*(number of tests)

*Although the ARTA–A method allows for up to 48 tests per run, *logistical considerations permit about 24 per Laboratory Technician*.

## Discussion

The exponential increase to nearly 15 million PLWHAs on ART by march 2015 and the shift from access to quality care, makes laboratory monitoring of HIV treatment increasingly important [1,2]. In light of the progressively shrinking donor basket, HIV treatment programs in RLS are expected to do more with less funding [15,16,17] and thus practical solutions to this problem are urgently needed. The VFA test was developed as part of an affordable HIVDR test algorithm, (ART–A). The VFA is unique, as it does not attempt to amplify target sequences irrelevant to HIV drug resistance monitoring in RLS (protease gene). Noteworthy, greater than 95% of PLWHAs in RLS are on first-line ART, which do not contain the Protease Inhibitors and are preserved for second-line in PLWHAs failing first-line Reverse Transcriptase inhibitor regimen. In addition, the VFA does not attempt to provide an absolute copy number of viral genomes/ml but rather focuses on a given threshold that is clinically significant (5000 or 1000 copies/ml).

We report a Viral Failure Assay on DBS that is practical, feasible, and highly specific, with moderate sensitivity, and is fairly accurate (9 out of 10 patients with VF were accurately identified). This VFA had a mixed performance with generally better overall results at a higher threshold of 5000 copies/ml. Nonetheless, the VFA had varying results with a higher sensitivity at a lower threshold of 1,000 copies/ml and at a higher specificity at a higher threshold of 5000 copies/ml. This variation in performance was also seen in adults compared to children, across HIV-1 viral subtypes, study sites and with ART status.

Although, the VFA overall performance was lower in terms of sensitivity and specificity than the gold standard, this shortcoming may be outweighed by the benefits of its feasibility and cost. The VFA performance could be considered in those situations where otherwise only clinical and immunological (CD4) monitoring would be available, which is suboptimal as compared to routine VL monitoring [18,19]. Further, the VFA showed a predictive value of positivity of 81.3% in the field among cohorts of PLWHAs on ART. Research literature demonstrates that CD4 testing has a low positive predictive value of viral failure [19]. It is thus prudent to suggest that the VFA provides an advantage in identifying PLWHAs on ART who require switching to 2^nd^ line therapy. The VFA generally demonstrated better receiver operating characteristics at a higher viral load threshold of 5,000 copies/mL, consistent with a recent systematic review [20]. In fact, this review showed that specificity was close to 100% at DBS VL above 5000 copies/ml, and this threshold would be the most reliable for predicting true virologic failure using DBS [20]. This puts into perspective the current definition of VF by WHO set at 1000 copies/ml [4] and questions whether this decision results in ineligibility of (promising) in house VL tests and in fact slows down roll-out of VL monitoring in RLS.

The finding that the VFA performed better in adults than children at our higher primary threshold of >5,000 copies/ml remains a paradox; particularly in light of the documented higher VL in Children as compared to adults [14]. A practical explanation could be that the volume and quality of DBS taken from children is less compared to adults suggesting an issue of volume input contrary to the viral load.

Our study was not without limitations. First the VFA performance was sub–optimal with lowering of the VF threshold to 1,000 copies/mL, increasing the possibility of false negatives. Hence, refining a simple technique for nucleic acid extraction from the DBS to allow maximum yield of nucleic acids, concentration of reagents, standardizing the volume of blood during sample collection could be considered in the future development of the VFA. These modifications would however be made at higher cost. Secondly, the VFA showed variability in performance across the Ugandan study sites, which highlights the need for further standardisation of the assay. Genetic variability is unlikely to explain these cross-site differences since Aitken and colleagues [7] previously optimized the assay for different HIV subtypes, which performed equally well across the various HIV-1 subtypes. Indeed there was no variation in the distribution of the HIV-1 viral subtypes across the study sites (55.2%, 55.8% and 61.4% for subtype D in Mbale, Mbarara and Kampala respectively) and among the samples that failed. Lastly, we did not consider maintenance costs, depreciation of equipment and costs due to electricity and waste disposal in our costing analysis. However, such costs would likely be much higher for the reference standard with bigger and more sophisticated equipment.

## Conclusions

The VFA demonstrated 89% of correct classification of the virologic response to ART in various field situations in Uganda. Basically, 1 out of 10 of patients were misclassified for VF, creating a potential risk of unnecessary or late switch to 2^nd^ line ART. Our findings present an opportunity to accelerate roll out of VL monitoring in RLS with an assay that produces results that are fully acceptable for public health application. Simple and low-cost routine virologic monitoring for HIV–1 treatment could prevent emergence of drug resistance in resource–limited settings. Future studies to refine the VFA performance would consider: developing an ideal reference assay for DBS; innovating a portable and solar powered automation with the potential to reduce the VFA turn–around–time and human error; as well as a full cost–effectiveness evaluation.

## Supporting Information

S1 Excel Supplementary file. (XLS)
